# Vulnerability, ageism, and health: is it helpful to label older adults as a vulnerable group in health care?

**DOI:** 10.1007/s11019-022-10129-5

**Published:** 2022-11-19

**Authors:** Elisabeth Langmann

**Affiliations:** Institute of Ethics and History of Medicine, University of Tübingen, Gartenstraße 47, Tübingen, 72074 Germany

**Keywords:** Vulnerability, Ageism, Healthcare, Olderadults, Ethics

## Abstract

Despite the diversity of ageing, society and academics often describe and label older persons as a vulnerable group. As the term vulnerability is frequently interchangeably used with frailty, dependence, or loss of autonomy, a connection between older age and deficits is promoted. Concerning this, the question arises to what extent it may be helpful to refer to older persons as vulnerable specifically in the context of health care. After analyzing different notions of vulnerability, I argue that it is illegitimate to conclude that older age is related to increased vulnerability. Much more, identifying older adults as a vulnerable group is closely related to ageism and can be associated with paternalistic benevolence and a tendency to overprotection, especially within health care. Additionally, even though older adults are more often in situations of increased vulnerability due to their potentially higher need for health care, I argue further that older adults mainly become a vulnerable group due to ageism. In this way, it can be concluded that the vulnerability of older adults does not originate in certain attributes of the group, but arises from a characteristic of society and, in turn, health personnel, namely ageism. Labeling older adults as vulnerable therefore is only helpful, when it is used to raise awareness of the widespread ageism in society, in this context, especially in the setting of health care, and the negative consequences thereof for older adults.

## Introduction

The connection between vulnerability and ageing is ubiquitous, complex, sometimes controversially discussed, and broadly addressed in public and academic debates. For instance, the common public narrative on ageing, being viewed as an inevitable process of decline, often portrays older adults as frail, vulnerable, and dependent (Centre for aging better 2021). Furthermore, older age is often perceived and presented as an economic, political, and social problem or even a burden (Makita et al. 2021). Especially during the Covid-19 pandemic, older adults were predominantly depicted as a vulnerable and homogeneous group in the media (Bravo-Segal and Villar 2020). Besides that, negative categorizations of older age and corresponding connections to vulnerability can also be widely found inter alia in the academic literature on health care. The consequences of such generalizations for the well-being of older adults can be manifold but are, in the context of vulnerability, mostly related to disadvantages in health care due to stereotypes, prejudice, or derived codes of conduct such as exclusion from medical research. This could be proven again just recently: Despite considering older adults, besides other persons with pre-existing conditions, as being the target group benefiting most from a Covid-19-specific vaccine, they were evidently underrepresented in the respective clinical trials (Prendki et al. 2020). Such observations make vulnerability a concept of special interest for the topic of older adults in health care and for the corresponding academic debates. Consequently, several conceptions of vulnerability exist in the literature, of which most are linked to embodiment, risks for well-being, and/or autonomy (Zagorac 2017; Bergemann and Frewer 2018). Due to the diverse interconnections of vulnerability to topics such as dignity, justice, benevolence, or non-maleficence, it is especially instructive to consider and include it within analyses of related phenomena and topics, such as older adults in the context of health (care), from an ethical perspective.

To overcome negative perceptions of ageing and older age and their potential consequences, the United Nations (UN) and the World Health Organization (WHO) jointly launched the initiative “decade of healthy ageing”. The overarching goal is to change how we think and feel about as well as act toward age and ageing (UN 2020; WHO 2021a). Therefore, challenging the understanding and conceptualization of vulnerability concerning older adults is a crucial issue, since labeling persons in a certain age per se as a vulnerable group can be problematic, even though older adults might very well be in situations, in which they become vulnerable. Consequently, important steps seem to be critically questioning the label of vulnerability and its background assumptions, also concerning age, the potential function or use of identifying a group (e.g., older adults) as vulnerable in health care, and possible connections with ageism. This entails the necessity to reflect on the (potential) paternalistic benevolence of the label of vulnerability and to address and, ideally, answer the question stated in the title of this article: *Is it Helpful to Label Older Adults as a Vulnerable Group in Health Care?*. For this, I intend to analyze associations and notions of older age and vulnerability and their possible implications. The first part of the paper aims to explore different understandings, concepts, and sources of vulnerabilities identified and available in the literature, especially in the context of health care. Thereafter, these understandings of vulnerabilities are contextualized within the topics of ageing and older adults. After analyzing to what extent the label of vulnerability might match, reflect, or fit understandings of older age, the ethical implications of such categorizations are elaborated to lay the groundwork for the argument in the second part of the paper. Therein, I will address vulnerability and older age in light of the concept of ageism, through which I will examine the hypothesis that due to the diversity of ageing and the negative consequences of categorizing a group as vulnerable, it is not helpful to label older adults as a vulnerable group per se. However, if older persons are considered vulnerable based on ageism, it can be argued that older adults are a vulnerable group due to the predominance of ageism. On the one hand, spelling out this line of reasoning can be helpful for future academic debates, addressing the corresponding topics in a clearer and more differentiated way. On the other hand, the findings can be helpful, especially for health professionals to be (made) aware of the phenomenon, interconnections, and implications of ageism. By acknowledging this, my argumentation and article aim to support combating ageism and creating an ageism-free health care system and society.

## Notions of vulnerability

A starting point for considering and reflecting on vulnerability can be found at the heart of various bioethical approaches, such as principlism or ethics of care: much of the corresponding analysis is built around potential risks to health and well-being, which can be in a certain sense understood as vulnerabilities (Rogers et al. 2012). Historically, in the context of health, vulnerability evolved as a term and concept to address and respond to significant malpractices within specific disciplines of research, namely medical and clinical research involving human subjects. Especially after World War II – in connection with the Nuremberg Code – the aim was to prevent inhumane experiments on particular groups of people, such as prisoners (Groß 2014). Only a few years later, the Declaration of Helsinki (DoH) was adopted, in which ethical principles for medical research on human subjects were elaborated (Wiesing et al. 2014). Over the years, the document has been revised several times, and vulnerability was only included for the first time in the fifth version (2000). In the subsequent revisions, the presented notion of vulnerability evolved from a reduced ability to give consent to the definition of a vulnerable group as exhibiting “an increased likelihood of being wronged or of incurring additional harm” and therefore stating that it “should receive specifically considered protection” (World Medical Association 2018). With such an understanding, vulnerability became relevant beyond medical research, especially to medicine, nursing science, and health care as such (Mergen and Akpınar 2021).

For decades, the approach of defining vulnerable populations was broadly used in health policies and led to many groups being labeled as vulnerable, such as also older people (Beauchamp and Childress 2019). Such a tendency for categorization promotes an understanding of homogeneity concerning the members of the respective group, which consequently bears the risk of stereotyping and prejudice. Furthermore, the label of vulnerability can have the effect of denying the respective group the capability of making their own decisions (ibid.), which can result in paternalistic benevolence or, in the case of clinical trials, potentially discriminatorily using it for or rather against their inclusion (CIOMS 2016). Critics argued that by labeling more and more groups of people as vulnerable without distinguishing specific characteristics, the concept might become vague, and through that can lose its utility (Levine et al. 2004). Besides that, the term vulnerability is frequently used but despite many efforts not consistently defined in health care (Clark and Preto 2018). In a recent systematic review of the literature on the concept of vulnerability in aged care, the meaning of vulnerability in the context of older adults and health has been analyzed from an ethical perspective. Thereby it is highlighted that vulnerability is value-laden and at the same time under-theorized as a concept (Sanchini et al. 2022). Much more, terms such as frailty, dependence, or loss of autonomy are often used interchangeably with vulnerability (Levasseur et al. 2022). Considering a prevalent understanding of autonomy common in the context of health care, in which persons are predominantly viewed as rational agents that can actively decide and therefore protect themselves, vulnerability is seen where autonomy (in this perception) is diminished or absent (ten Have 2016). In that regard, incapability of making (informed) decisions or being able to protect oneself from harm contributes to the contextualization of vulnerability (Mergen and Akpınar 2021), but at the same time is ascribed to those, who are labeled as vulnerable. Such an undifferentiated view, in which vulnerability is interpreted as a lacking capacity “to make informed judgments for oneself, being socially or economically disadvantaged, or […] the result of other factors that contribute to a lack of autonomy” (Nuffield Council on Bioethics 2007), is pervasive. In particular, the literature identifies incapability of self-determination as a condition of vulnerability (Sanchini et al. 2022). In turn, this understanding reflects a mostly deficit-oriented view of the topics concerned and an individualistic perception of autonomy.

However, since the notion of vulnerability has been on the agenda of feminist theory (from around 2010 onwards), not only has the research interest and focus increased but also the concept has developed significantly (Mergen and Akpınar 2021). For example, Mackenzie et al. explored the phenomenon of vulnerability in a more differentiated way by proposing to focus on sources of vulnerabilities instead of using the term as a label for groups of people (Rogers et al. 2012; Mackenzie et al. 2014; Mackenzie 2014); specifically, two basic sources of vulnerabilities are addressed.^1^ The first one is based on the human condition (as embodied and social beings), making people per se inherently vulnerable (to wounding/suffering) (Mackenzie 2014). Such ontological vulnerability and the corresponding sources are understood as *inherent* to all humans. This is linked to the possibility of disease and sickness, as well as the inescapability of death and dying. It is through such vulnerability as a *conditio humana* that also the human rights, and thus specifically the right to health, become a necessity (Bergemann 2018). Furthermore, in such a view, as social beings, we are dependent on the actions of others, which makes us vulnerable to them as well and directly links the concept of vulnerability to dependency. In this context, Mackenzie highlights factors that may influence inherent vulnerabilities, such as age, health status, or gender. She mentions “ill health” or “extremes of age” as “creating” new vulnerabilities or “exaggerating” existing ones (Mackenzie 2014).

The second source of vulnerability Mackenzie discusses is (more) context-specific and “focuses on the contingent susceptibility of particular persons or groups to specific kinds of harm or threat by others” (ibid.). Consequently, people are (particularly) vulnerable due to certain, e.g., personal, socioeconomic, or environmental factors/situations and, thus, have “reduced capacity, power, or control to protect their interests relative to other agents.” (ibid.). Mackenzie et al. (2014) define such sources of vulnerabilities as *situational*, which they also extend with the subset of *pathogenic* vulnerabilities. In the strand of literature discussing vulnerabilities as situational, Luna (2009) proposed a dynamic conception of vulnerability as existing in layers, which can be understood as relational and dynamic. Therefore, multiple kinds, sources, and even overlaps of vulnerabilities can be interpreted, each being connected to specific situations and contexts, e.g., informed consent or social circumstances (ibid.). Through such an understanding, the concept of vulnerability gains in flexibility and discreteness while, at the same time, helping to avoid falling for generalizations and stereotypes. Furthermore, this picture of layers makes the concept of vulnerability more dynamic by allowing for vulnerabilities to operate in parallel.

As a subset of situational vulnerabilities, according to Mackenzie, the above-mentioned pathogenic sources can be understood as a powerful tool to highlight vulnerabilities arising, in particular, from ethically concerning contexts such as “morally dysfunctional or abusive interpersonal and social relationships, and sociopolitical oppression or injustice” (Mackenzie 2014). This source of vulnerability is closely related to the unequal distribution of power in interpersonal relationships and often results from stereotypes and prejudices.

Pathogenic vulnerability also exists, when an action aims to improve vulnerabilities, but paradoxically worsens them. This may particularly be the case, for example, in health care. Here, situational vulnerability may already exist due to health status or the situation of dependency, in which an unequal power relationship may exacerbate vulnerabilities. One characteristic of this is that in such situations, autonomy is undermined or the feeling of powerlessness is intensified, which in turn increases vulnerability. Health care institutions can thus be places of pathogenic vulnerability, which necessitates particular caution when addressing vulnerabilities in this context. Thereby, pathogenic vulnerability stands for unacceptable occurrences in situations of dependence. By becoming aware of this, this tool and concept increases the chance of ameliorating harm.

The presented classification of vulnerability might become clearer with the help of the following example: A person, who visits the hospital for treatment of an illness, finds herself situationally vulnerable, firstly, due to the need for health care and secondly, due to the prevailing dependence on the health personnel. If in that situation, paternalistic decisions concerning treatment are made (e.g., by the health personnel or relatives) – also from the idea and with the intention of (good) care – pathogenic vulnerability occurs as a result of the existing unequal power relations. Thus, health care institutions can be sites of pathogenic vulnerability by, among other things, undermining patient autonomy, inadequately addressing (other existing) needs, and/or exacerbating feelings of powerlessness and loss of control (Mackenzie 2014). In this regard, Mackenzie argues that viewing vulnerability and autonomy as opposing concepts cannot be beneficial for health care that aims to meet the needs of individuals (ibid.). With the idea of beneficence in mind, this would create much more the risk of paternalistic relationships between health care professionals and the patient. To avoid this, it is fundamental not only to respect and promote self-determination but also to place the autonomy of the person at the center of care, whereby interventions can be implemented that promote autonomy and *at the same time* minimize vulnerabilities. This contradicts the above-mentioned individualistic notion of vulnerability as a lack of autonomy. Additionally, if autonomy is understood relationally, a seemingly existing contradiction between protection against vulnerabilities and autonomy is dissolved through a non-paternalistic form of safeguarding (ibid.). In addition to illustrating the presented classification of vulnerability, the example also shows, in a broader sense, the relational nature of vulnerability. While the described vulnerabilities exist in this very context, they do not necessarily persist beyond the specifics of the situation, which means that as the situation changes, also the existing vulnerabilities can change or even vanish. Possibly, the affected person can even no longer be understood as vulnerable (Luna 2019). This is only conceivable based on a dynamic conception of vulnerability that is not understood as implying a categorical approach. It is also only against this background that it seems possible to combat context-specific vulnerabilities through concrete measures. Luna (2015; 2022) even points out that due to the varied nature of vulnerabilities, they defy orderly classification, whereby categorizations bear the risk of introducing rigidity that does not reflect reality in this context. Although the taxonomy developed by Mackenzie et al. is useful for identifying and sorting different sources and causes of vulnerability, a more flexible approach, such as the metaphor of layers proposed by Luna, seems more adequate in practice.

## Views on vulnerability concerning ageing

Often older age and vulnerability are understood as being inherently linked to each other and, sometimes, older adults are even described and handled as a paradigmatic example of vulnerability; the main reason presented to explain this presumed connection is biological and cognitive decline in the context of ageing being connected to a higher risk of diseases (ten Have 2016). This is associated with an understanding that assumes a progressive loss of power and control when becoming older (Sanchini et al. 2022). This view became also evident through a large-scale survey, in which especially younger people agreed to the statement that older age is characterized by frailty, vulnerability, and dependency. In contrast, older participants tended to reject this understanding, with just under a third of the oldest (70+) even stating that we should not expect a physical and cognitive decline in older age (Centre for aging better 2021). Although there is increasing effort to highlight the many faces of growing older and also its positive effects, the common deficit-oriented view of older adults was clearly evidenced during the Covid-19 pandemic (Ayalon et al. 2021). Especially, official as well as media communication often categorized seniors practically definitively as vulnerable (Bravo-Segal and Villar 2020) and inter alia urged older adults quite generally to limit their social contacts during the pandemic. At the same time, particularly in the context of possible triage criteria for emergencies, there was an open discussion about the chronological age of patients and the extent to which this should be taken into account when prioritization decisions in hospitals are needed (Previtali et al. 2020; Ehni and Wahl 2020). As a result, the following two main narratives have been identified. First, the “vulnerability narrative”: as it became clear that with higher age the risk of severe illness and mortality due to Covid-19 increases, older adults were portrayed as a highly homogeneous and vulnerable group. Second, the “burden narrative”: before long, older adults were portrayed as a burden to society and particularly in connection with an overwhelmed health care system and the increased risk of triage in hospitals (Ayalon et al. 2021). Thus, not only the vulnerability ascribed to older adults by society became once again visible through the pandemic, but also the widely spread homogeneous and deficit-oriented view of ageing. Furthermore, the already mentioned interchangeable use of the terms vulnerability and dependence, frailty, or decline of autonomy does not only show the conceptual confusion but these presumed synonyms also illustrate the context in which the term vulnerability is understood and operates. To further analyze how and if the term and concepts of vulnerability can (indeed) possibly be helpful concerning older adults, the notions and different definitions of vulnerability as described earlier shall be contextualized with the topics of ageing and older age in the following in more detail.

Ageing and associated changes are complex, multifaceted, and relate to diverse aspects of human existence, which is why it is also a highly interdisciplinary research topic. From the perspective of health research, ageing can, inter alia, be described as a process that increases the likelihood of health modifications, which can also be correlated with diseases. However, it needs to be emphasized that ageing is not a linear process but proceeds in a myriad of ways and is influenced by many dimensions such as genetic factors and socioeconomic status, which can themselves be interconnected in various highly complex ways (Tesch-Römer 2019; Apóstolo et al. 2018). In conclusion, ageing persons will experience vastly different health developments, especially as a function of their chronological age. Therefore, even though chronological age is information that is easily accessible, it is problematic to use it as a direct indication of health status. Apart from that, many age-related physiological changes, such as a decrease in vision, can be understood as “side effects” of the ageing process, which are perceived as limitations oftentimes only due to a lack of support. Accordingly, such age-related physiological changes are not to be regarded as diseases per se but depend on their context. However, they can (co-)shape the understanding of health and disease in old age. However, it should be highlighted that despite an increased likelihood of chronic diseases in old age, the “elderly”, as they are often referred to collectively, represent a very heterogeneous group in society that cannot be adequately described by generalized statements about their health status. This heterogeneity is reflected in the following: the prevalence of limitations in everyday life increases with age. For example, a survey in all EU countries showed that about 45% of people 75 or older experience limitations in coping with everyday life (OECD 2020). This shows that besides a number of older adults living with limitations or diseases, many live their everyday lives actively and healthily. Therefore, it should be emphasized that being old does not necessarily mean being ill (Schwartz and Walter 2016), nor does it mean that you cannot be involved in communities and society at large. Moreover, for example, living with chronic illnesses does not per se prevent (older) people from living a “normal” life or, more generally from being able to perform the activities of daily living.^2^ Therefore, undifferentiated understandings and definitions of vulnerability (e.g., connected to criteria such as “lacking the capacity to make informed judgments” (Nuffield Council on Bioethics 2007)) cannot be used to ascribe vulnerability to (all) older adults, and chronological age can certainly not be regarded as the reason for such vulnerability. Moreover, even if quite a number of persons in a group such as older adults could be identified as vulnerable (following certain understandings/arguments), keeping in mind the diversity of the group (specifically in the context of ageing), a lot of them might not be particularly vulnerable. Further, if all older persons are labeled as vulnerable (erroneously), not only does the label become vague, but it could lead to overlooking those who might indeed need special protection. Concerning this, a qualitative study including 222 participants on the perception of vulnerability concerning older adults found that chronological age was only associated with vulnerability by 2% of the interviewed, much more, vulnerability was directly linked to diseases (Bajotto et al. 2017). Furthermore, the deficit-oriented view on ageing, which also becomes apparent in the above-mentioned understanding and definition of vulnerability, does not reflect the various lives of older adults, but blurs the diverse realities of these people. Such a view conveys stereotypes via attributions of “age-appropriate” abilities and skills, which can result in prejudice and induce unfair treatment, disadvantage, or even discrimination in a wide variety of ways (Chang et al. 2020), as described in the next section of this article. For these reasons, among others, it seems to be important to challenge the understanding of vulnerability as a label that refers to a lack of autonomy and loss of agency. Moreover, the predominantly rationalistic and individualistic perception of autonomy transported by the above-mentioned pervasive views is criticized also due to its implications on vulnerability, especially from a feminist perspective (Rogers et al. 2012; Mackenzie 2014; Luna 2009). In the following, possible and presumed vulnerabilities of older adults within different understandings of vulnerability, as discussed in Sect. 2 of this article, are critically analyzed.

With regard to the understanding of vulnerability as “lacking the capacity to make informed judgments for oneself” (Nuffield Council on Bioethics 2007), older adults cannot be categorized as such, especially due to the diversity of ageing. Accordingly, it cannot be assumed that older persons can be understood as a vulnerable group or, effectively, vulnerable per se; nor can such an interpretation be considered helpful as it does not follow a sufficiently adequate view of older adults and their autonomy and as it promotes paternalistic benevolence. In line with this, Bozzaro et al. (2018) also conclude that “older age cannot be considered a general marker of vulnerability”, and doing so would be problematic based on the following arguments: the interconnectedness of vulnerability with negative stereotypes of ageing and the interpretation of being vulnerable as a lack of autonomous agency; additionally, it is also pointed out that ageing cannot be understood as a constant state but rather a diverse process (ibid.).

However, if vulnerability and age are analyzed according to the taxonomy by Mackenzie et al. (2014), different sources of vulnerabilities in connection with age become apparent, making them also more easily relatable to each other. First, as outlined concerning inherent vulnerability, every person can be understood as vulnerable. Older age may, prima facie, be associated with vulnerability, due to a higher risk of illness and chronic diseases. As mentioned above, Mackenzie highlights age and health status as factors that may influence inherent vulnerabilities. For instance, “ill health” or “extremes of age” might “create” new vulnerabilities or “exaggerate” existing ones (Mackenzie 2014). In this context, Turner (2006) even directly connects inherent vulnerability with ageing, already on a theoretical level, by stating “ageing bodies are subject to impairment and disability”. As stated above, it appears to be, nevertheless, illegitimate to conclude that older age is in particular and in general connected to an increased inherent vulnerability, referring to the plethora of ways in which people live and age. Especially problematic is an inference from a certain statistical correlation to a single person’s vulnerability (with its consequences), which is very much dependent on individual factors that (also statistically) influence the personal ageing process to pan out in vastly different manners. Despite age not being at the center of Mackenzie and colleagues’ analyses, a generalizing undifferentiated and deficit-oriented view on ageing becomes visible through the association of (older) age with increased inherent vulnerability on these premises. Although the risk for illnesses or diseases might correlate with (chronological) age, furthermore, the plethora of mediating variables and other factors influencing this relation make it, in conclusion, questionable to state in general terms (based on these grounds) that a person at age X is more vulnerable than a person at age X-1.

Second, in terms of health and older age, older adults may be more often in contexts of situational vulnerability due to their potentially greater need for health care. Therefore, they might be on average more often confronted with *situational* vulnerabilities but cannot per se be regarded as more situationally vulnerable analogous to the argumentation for inherent vulnerability above. This means, although in many cases older people make greater use of health services than younger people, it cannot be concluded that this results in a higher vulnerability. In this context, Bergemann (2018) acknowledges and highlights the importance of vulnerability-sensitive health care, which he describes as mindful and person-oriented. However, since vulnerability is predominantly characterized as a deficit, coining person-oriented health care as vulnerability-sensitive can be misleading. Thus, the argument can be better met by calling for health care that focuses on meeting the individual needs of people. Nevertheless, vulnerability-sensitive care could indeed be valuable in contexts of situational vulnerability by consciously addressing such sources and accordingly reducing them, additionally, avoiding pathogenic vulnerabilities (see below). In relation to that and concerning the understanding of vulnerability as existing in layers, as Luna (2009) proposes, it is not helpful to label certain groups per se as vulnerable, but rather pay attention to particular situations that may add layers of vulnerability, through which certain persons become vulnerable in specific contexts. Consequently, if a situation of such vulnerability changes, the persons in question may no longer be considered vulnerable. For example, thinking in layers could mean that if the situation of the needs of older persons in health care is adequately addressed, it can be argued that being older does not, in itself, imply vulnerability. A relevant example seems to be the layer of vulnerability concerning physical health. In case of older age and functional limitations, such as reduced mobility, a respective vulnerability might manifest when healthcare institutions are not accessible without barriers. But if, for example, an older person who is equipped with an appropriate walking aid, heads to the pharmacy and finds adequate infrastructure, such as ramps, the (potential) vulnerability dissolves. This illustrates that vulnerability (e.g., due to functional limitations) can exist, but can also vanish depending on the specific facets of a situation. Layers of vulnerability can include social and economic circumstances, such as relationships and social participation, but also financial situations in addition to health-related aspects. All these situations of vulnerability can and might more likely be encountered in older age, but *cannot* be understood as vulnerabilities *of* older age. Much more, with older age, diverse layers of vulnerability might reinforce one another, through which older adults are at risk to become more vulnerable, as Luna (2014) points out, due to missing efforts and policies to prevent and act against them.

This is related to a pathogenic source of vulnerability, which, similar to all situational vulnerabilities, older adults may be at higher risk of being confronted with, due to various but especially due to ageist reasons. This relation makes ageism (one of) the central topic(s) concerning the vulnerability of older adults. Overall, the concept of layers seems to be especially helpful concerning different shades of vulnerability (also in this context) and appears to simplify the consideration of intersectional aspects in a corresponding analysis, which can be of particular importance regarding ageing and ageism.

## Ageism and vulnerability

Ageism can be defined as negative or positive stereotyping, prejudice, and/or discrimination against older people based on their chronological age or the perception of them as being “old” or “older” (Iversen et al. 2009). Hostility towards older adults can thus exhibit cognitive, affective, and behavioral elements and can be implicit or explicit. It involves how we think and feel about, as well as act toward older persons based on chronological age or age classification (ibid.). As stated in the “Global Report on Ageism” by the WHO, at least every second person worldwide is ageist towards older adults; additionally, every third older person (in Europe) has already experienced ageism, making billions of people affected (WHO 2021b). Therein, views on ageing, as individual and societal conceptions of ageing and of being old, play a central role, depicting stereotypes that can be both positive and negative (Wurm et al. 2020). As age is one of the first things we notice about people, the vagueness of this label is often not adequately taken into account in the following considerations. We assign “age-appropriate”’ characteristics to persons and develop views on ageing that are shaped by subjective and social attitudes, preferences, etc. Such notions and thus the categorization of persons in older age are predominantly based on prejudices, whereby they are not only descriptive but much more normatively effective and can result in ageism. Consequently, such categorization can be used to remove a layer of vulnerability and therefore sensitive behavior towards persons or groups, but can also, as mentioned earlier, result in unfair treatment, disadvantage, and discrimination in a wide variety of ways (also ageism) (Ayalon and Tesch-Römer 2018).

In the context of health, ageism can be widely associated with poorer health status. This correlation is the result of, e.g., denied access to health services and treatments or the partial exclusion of older persons from health research (Chang et al. 2020). An illustrative example of this is research into Parkinson’s disease, in which almost half of the studies were conducted without the participation of older people (Fitzsimmons et al. 2012).

Similarly to the general categorization of older adults, associating older age with vulnerability can be used for sensitive behavior toward older adults, or it can also support negative views on ageing, reduce the autonomy of older adults through paternalistic benevolence, and therefore provoke, inter alia, pathogenic vulnerabilities. All of this can lead to negative health consequences for the affected. A clear example of a pathogenic vulnerability in health care is the undermining of personal authority via the use of language, specifically concerning how medical staff speaks to older people in the context of treatment. If older patients are generally spoken to at a slower pace of speech and in simpler sentence structures due to existing age prejudices, this not only resembles communication with children but also presupposes a person’s needs without having asked for them. Psychologist and expert on person-centered communication Storlie even names ageism, or language influenced by ageism, as the biggest obstacle to good communication (Storlie 2015). Although interpersonal communication is always a challenge, especially in the area of health care, insufficient exchange also has a potentially (direct) negative impact on the respective well-being. This is not necessarily caused by ageism, but is exacerbated by it. Accordingly, the use of so-called “elder speak” can be perceived and described as disrespectful and condescending to the person in question. Even if good intentions are at the forefront, “elder speak” can, inter alia, lead to isolation, depression, or a feeling of reduced control for the affected person (Swift et al. 2017).

Such and similar negative experiences in helping institutions, such as clinics, can affect the respective persons in a way that they consequently try to avoid both health institutions and health personnel. On the one hand, this may be more of a potential consequence, so that, in the case of illness, more effort is required to seek help; on the other hand, it may lead to active avoidance of health care institutions, which represent sites of experienced injustice, with the risk that by not using health services, people are actually harmed by their negative experiences. In sum, this is an example, in which older adults are understood as vulnerable due to (implicit) ageist stereotypes and, therefore, health personnel interact with them paternalistically and not adequate to their needs. Through this, a pathogenic vulnerability arises due to ageism, which, in this case, can have direct consequences on the well-being of older adults. Thus, if older persons are considered vulnerable based on ageism and (even only potentially) negatively affected by corresponding behavior, it can be argued that older adults *are a vulnerable group due to the layer of ageism being prevalent in society.*

In this way, it can be concluded that the vulnerability of older adults does not originate in certain characteristics of this group of people (such as frailty or risk for diseases), but arises from a characteristic of society and, in turn, health personnel, namely ageism. Vulnerability can thereby be understood not as a label for older adults, but much more as a warning sign for everyone concerning the avoidance of implicit and explicit forms of ageism. Labeling older adults as vulnerable therefore is only helpful, when it is used to raise awareness of the widespread ageism in society, in this context, especially in the setting of health care, and the negative consequences thereof for older adults. A result of such reasoning could indeed be the call for vulnerability-sensitive care, in which signs of ageism and thus situational vulnerability are recognized and consciously addressed through nothing but health care that focuses on the actual individual needs of persons – no matter the age. In this context, the generation and application of geriatric knowledge play a central role, which can only be followed by evidence-based and thus safe care and the recognition of special needs. Especially when multiple diseases exist at the same time and thus care becomes more complex, the inclusion and consideration of individual life concepts in therapy planning are indispensable. Particularly against the background of the wide range of ageism, it must be emphasized that this does not translate to age-specific, but much more needs-oriented care in older age. This means that especially complex clinical cases associated with older age must be researched specifically, or the transferability of research results to different contexts must be scrutinized before concrete application. Ensuring evidence-based health care is thus not only central to professional action in the care of older patients but also fundamental to the well-being of older persons and thereby ensuring their right to health. In consequence, combating ageism is a means to reduce the vulnerability of older adults via sensitizing to the interconnections of stereotypes, prejudice, and discrimination concerning older age and health. Among these, a major challenge is to neither relativize nor deny possible negative aspects of ageing, but to recoin the views on ageing according to its many different ways. By highlighting the diversity and promoting positive narratives on ageing, not only more realistic views can be established but also an understanding, in which ageing is seen as a process of change with equal value.

## Conclusion

In this article, it was shown that older persons are often labeled and referred to as a homogenous and vulnerable group. Through looking at different common understandings of vulnerability and the diversity of ageing, it can be argued that older persons are not a vulnerable group per se. However, the presented approaches of sources and layers of vulnerabilities bring light to various relevant aspects of vulnerability, also in the context of older adults in health care, that can be otherwise overlooked. Consulting the layered approach, factors can be analyzed and identified that have complex interconnections and sometimes even operate in parallel. For example, ageist stereotypes have a close relationship with labeling older adults as vulnerable. Thus, by labeling older adults as such, ageist stereotypes are promoted, which can lead to ageist consequences in health care, and thus have critical normative implications.

Overall, with this analysis, it could be demonstrated that a clear-cut categorization of older persons as vulnerable is not only undifferentiated but can even do harm by provoking such negative age associations. Labeling certain groups as vulnerable is often accompanied by paternalistic benevolence and overprotecting attitudes that can lead to (unintentional) stereotyping and discrimination, which was shown within the analysis of pathogenic vulnerabilities. On that basis, it can be concluded that older adults are indeed a vulnerable group, but not in the common understanding but rather due to ageism. Thus, ageism adds a layer of vulnerability to the affected persons, in this case indeed to older adults as a whole. This means that even though they do not represent a (homogeneous) group based on their common age, older adults are being treated as one on the premises of ageism and thus are collectively disadvantaged. In this context, it is possible to formulate such a group-specific statement due to ageism being such a widespread and multifaceted phenomenon. Therefore, vulnerability based on ageism, as presented here, can look like a label, but is indeed still a layer that can vanish if society changes and combats ageism successfully.

In conclusion, it is *not* helpful to understand older age as vulnerable, neither for older adults themselves nor for health professionals. Becoming aware of the often mistaken label of vulnerability and its potentially harmful consequences may support overcoming a layer of vulnerability. Concerning this, the need for combating ageism actively and raising awareness of the diversity of ageing becomes clear. Accordingly, the label of vulnerability in that specific context can be helpful when it refers to the negative effects of ageism in health care and aims to be sensitive to the phenomenon and contributes to combating it. Nevertheless, using the term vulnerability can also in this context easily be misinterpreted, through which it can be recommended that the terms “vulnerable” as well as “vulnerability” should be avoided when speaking about or with older adults.

In terms of implications for health care practice, the argumentation of this article stimulates the following calls for action. First and foremost, it is imperative to combat ageism within and outside of health care institutions. As part of this, rethinking society’s perception of older adults and recognizing the heterogeneity of older age becomes necessary, as well as sensitizing and creating awareness of the far-reaching negative effects of ageism. Specifically, studying the phenomenon of ageism needs to be included in curricula of education and in further training programs in the health sector. Furthermore, different structural conditions and processes that may hinder persons with more complex health conditions in their claim for their right to health need to be examined, reviewed, and improved. This also includes the pursuit of non-paternalistic forms of health care as well as protection against pathogenic forms of vulnerabilities and thus promoting autonomy also in situations of needed assistance and care. Besides investigations into the broad dynamics and implications of ageism, further research should be done concerning paternalistic benevolence, resulting from prevalent understandings of older persons’ vulnerabilities and their implications for autonomy and self-determination. Especially considering the widespread deficit-oriented views on ageing, it also seems to be of great importance to examine how ageism and ableism are intertwined in health care and how this impacts the notion of vulnerability.

## Footnotes

1 Subsequently, the taxonomy/nomenclature of Mackenzie et al. is used.

2 Activities of daily living are central activities for fulfilling basic physical and mental needs. These include personal hygiene, communicating, and sleeping.
